# Complete Proton Transfer Cycle in GFP and Its T203V and S205V Mutants**

**DOI:** 10.1002/anie.201503672

**Published:** 2015-06-18

**Authors:** Sergey P Laptenok, Andras Lukacs, Agnieszka Gil, Richard Brust, Igor V Sazanovich, Gregory M Greetham, Peter J Tonge, Stephen R Meech

**Affiliations:** a School of Chemistry, University of East Anglia Norwich, NR4 7TJ (UK); b Department of Chemistry, Stony Brook University Stony Brook, NY 11794-3400 (USA); c Central Laser Facility, Research Complex at Harwell, Harwell Science and Innovation Campus Didcot, Oxon OX11 0QX (UK); d Department of Biophysics, Medical School Szigeti str. 12, 7624 Pécs (Hungary); e The Scripps Research Institute, 130 Scripps Way Jupiter, FL 33458 (USA)

**Keywords:** green fluorescent protein (GFP), IR spectroscopy, kinetic isotope effect, proton transfer, ultrafast spectroscopy

## Abstract

Proton transfer is critical in many important biochemical reactions. The unique three-step excited-state proton transfer in avGFP allows observations of protein proton transport in real-time. In this work we exploit femtosecond to microsecond transient IR spectroscopy to record, in D_2_O, the complete proton transfer photocycle of avGFP, and two mutants (T203V and S205V) which modify the structure of the proton wire. Striking differences and similarities are observed among the three mutants yielding novel information on proton transfer mechanism, rates, isotope effects, H-bond strength and proton wire stability. These data provide a detailed picture of the dynamics of long-range proton transfer in a protein against which calculations may be compared.

The green fluorescent protein from *Aequorea victoria* (avGFP) exhibits excited-state proton transfer (ESPT), in which a proton is transported ca. 10 Å down a “proton wire”.^1^ This unique property has established GFP as a model system to study proton transport in biology.^2^ The vast majority, though not all,^3^ of the time domain data on avGFP concerns only the ultrafast ESPT step.2d, 4 Here we characterize the complete proton transfer cycle in real-time using femtosecond to microsecond time resolved infrared (TRIR) spectroscopy. Measurements on avGFP are compared with two mutants, T203V and S205V, which modify the structure of the proton wire and thus the proton transfer dynamics.

In wild-type avGFP the chromophore exists in two charge states at physiological pH, the neutral protonated A state and anionic deprotonated B state.1a,c Time resolved fluorescence showed that the two charge states are connected via ESPT, where excitation of the blue absorbing A state leads to green emission from an I* state within a few picoseconds, where I is the chromophore anion in the environment of the A state; I and B are connected by a rare reorganization.2d Structural studies showed that the likely proton acceptor is an ionized glutamic acid residue (E222) connected to the photolabile proton through a proton wire comprising a conserved water molecule and S205 (Figure 1).^5^ Thus, avGFP is a unique example of long-range proton transfer in a protein that can be optically initiated and so studied by time resolved spectroscopy. That E222 was indeed protonated (and on the same timescale as A* decay and I* formation) was shown by TRIR spectroscopy.4a Subsequently experimental and theoretical methods were deployed to probe dynamics on the proton wire, and a detailed picture emerged of proton transfer rates, wire dynamics and structure.2b,c,e, 4b,c, 6

**Figure 1:**
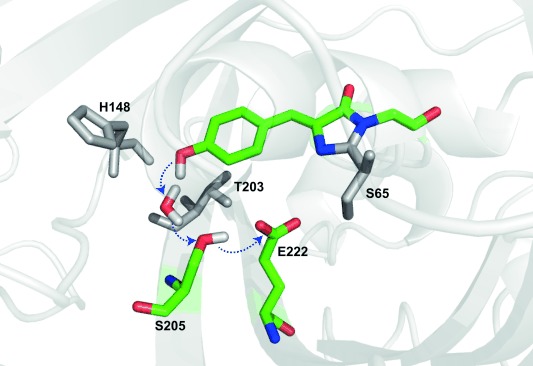
Structure of wtGFP proton wire (1GFL pdb). The residues in the proton wire (colored) and key surrounding residues (gray) are highlighted.

Particularly informative were mutations in the vicinity of the proton wire (Figure 1). Both the S65T mutation and replacement of E222 disrupt the proton transfer chain, so no I* emission occurs on excitation of A; however, a further mutation (H148D) rewires proton transfer, via a short low-barrier H-bond to D148, to which ESPT occurs on a sub-picosecond time scale.2a, 7 In contrast, mutations T203V and T203Y shift the emission of I*, but do not significantly alter ESPT. Surprisingly, the S205V mutation, directly in the proton wire of avGFP, does not eliminate ESPT, but slows it by up to 30-fold. X-ray data (Supporting Information, Figure S1) suggested a new proton wire is formed by a water molecule, T203 and E222.^8^ The slow ESPT in S205V has been studied extensively by fluorescence and simulation.^8^, ^9^

The dominant fate of I* is fluorescence to I and subsequent repopulation of A, completing the photocycle. The repopulation is not usually observed, as ground state proton transfer (GSPT) is faster than the nanosecond I* decay. Uniquely, Kennis et al. used ultrafast pump–dump–probe spectroscopy to populate the I ground state by stimulated emission, and showed that GSPT is biphasic and slower than ESPT.^3^ They also discovered a large kinetic isotope effect (KIE), where GSPT in D_2_O was longer than the upper time limit of the ultrafast experiment (>5 ns compared to 400 ps in H_2_O).2d Intriguingly, this result suggests that an experiment with sub-picosecond to microsecond time resolution could resolve the entire photocycle of avGFP in D_2_O. A recent experiment combines the vibrational and temporal resolution of TRIR with greatly extended observation times.^10^ Here we apply that method to record the complete photocycle of avGFP, T203V and S205V. We assign the marked differences in kinetics between ESPT and GSPT, characterize the novel pathway in S205V and quantify the KIE in each mutant.

The TRIR data for T203V and S205V are shown in Figure 2 on the picosecond to 0.1 microsecond timescale. The A state was excited at 400 nm with a 100 fs pulse and the response probed between 1300 and 1800 cm^−1^. Sub-nanosecond TRIR data for T203V (Figure 2 A) are very similar to those for avGFP (see Ref. 4a, 6 and Figure S2). The negative signals (bleaches) appearing at time zero are due to vibrational modes of the chromophore ground state, removed or shifted on excitation, and were assigned to C−O (1681 cm^−1^), C−C (1642 cm^−1^), phenyl ring localized modes (e.g. at 1592/1596 cm^−1^) and to vibrations of the protein perturbed by excitation.^6^ The positive signals (transients) are due to the excited state of the chromophore and perturbed protein modes. The time-dependent growth of the transient at 1710 cm^−1^ arises from protonation of E222 via the proton wire. The increasingly negative features developing after *t*=0 at 1560 and 1442 cm^−1^ on the same timescale as the growth at 1710 cm^−1^ are due to the disappearance of symmetric and anti-symmetric modes of the carboxylate form of E222.4c, 6 Both transient and bleach kinetics are non-single exponential and similar to the fluorescence decay; the kinetics, spectra and KIE for T203V are very similar to those of avGFP (Figure S2).

**Figure 2:**
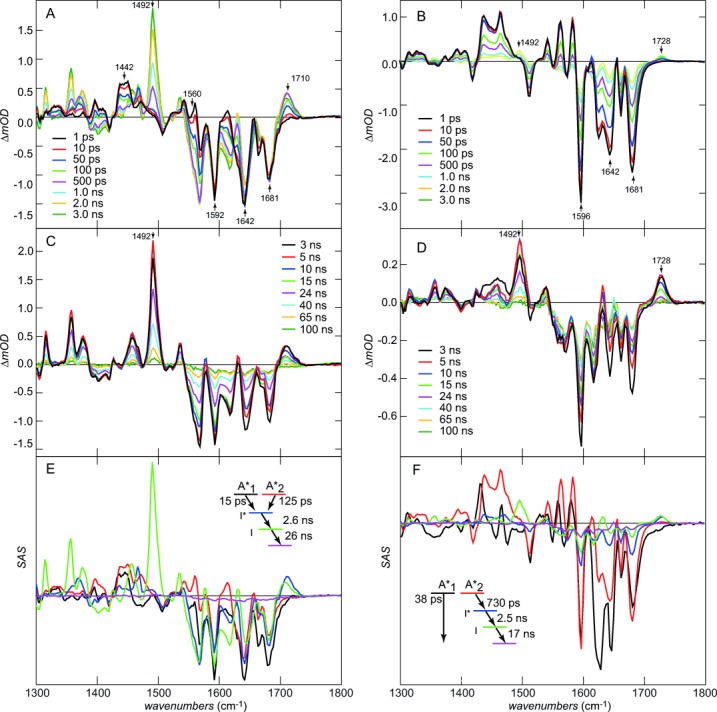
Picosecond to microsecond transient IR data (equivalent data for avGFP are shown in the Supporting Information). All samples in deuterated buffer. A) 0–3 ns IR difference spectra for T203V. B) Same as (A) for S205V. C) 3–100 ns IR difference spectra for T203V (the modes associated with I are highlighted). D) Same as (C) for S205. E) Species associated spectra for the T203V photocycle. The inset shows the kinetic scheme. F) Same as (E) but for S205V.

In contrast, Figure 2 B reveals important similarities and differences between ESPT in T203V (or avGFP) and S205V, in which the proton wire has been rerouted (Figure 1 and Figure S1). The most significant similarity is the rise at 1728 cm^−1^ in S205V, which is assigned to protonation of E222, providing spectroscopic evidence that the two proton wires have a common terminus, even though the rate of ESPT is greatly reduced (Figure S3). That this mode appears at higher wavenumber in S205V suggests that the H-bond between E222 and T203 is weaker than that between E222 and S205 in T203V. Significantly, in addition to the slower ESPT, S205V has a lower apparent yield of I* formation (compare the relative amplitudes of the *t*=0 phenyl ring bleach at 1592/1596 cm^−1^ to the peak of the E222 transients after 1–3 ns). There are a number of possible reasons for a lower yield, including kinetics (slow formation of I* in S205V in competition with the ca. 3 ns I* decay) and spectroscopy (the transition moment may be lower). However, TRIR data for S205V also reveal a rapid ground state recovery not present in T203V; compare the kinetics at 1592/1596 cm^−1^, which hardly recovers at all in the first 100 ps in T203V, but show strong recovery in S205V (Figure 2 A,B and Figure S4a). We can thus assign the relatively low yield to a population of S205V where the proton wire is not properly formed, so the ESPT cannot occur; this population instead relaxes directly from A* to A. The existence of such a channel is confirmed by time-resolved fluorescence up-conversion, where a 9 ps component in the decay of A* which does not appear in the rise of I* was resolved (Figure S3 b). The fast quenching may be associated with intramolecular motion of the chromophore unconstrained by formation of the proton wire; such flexibility promotes rapid internal conversion in the FP chromophore.^11^

There are further differences in the sub-nanosecond spectra of S205V and T203V, most notably in the complex bandshape around 1350–1450 cm^−1^, which contains contributions from both the chromophore excited state and perturbed carboxylate modes of E222.4c These differences also suggest a modified distribution of H-bond strengths in the two proton wires.

Figure 2 C and D show the photocycle measured on the nano-to-microsecond timescale. The most striking feature in T203V is the appearance in a few nanoseconds of an intense transient at 1492 cm^−1^, which decays in tens of nanoseconds leading to essentially complete recovery of the initial A state. This mode is readily assigned to formation of the anionic I state, as it corresponds with the most intense bleach observed in TRIR of the B state of S65T (Figure S5); indeed other bleaches associated with the B state are identified as transients in Figure S5. This chromophore anion mode is observed in D_2_O for both T203V and S205V but not in H_2_O, consistent with the fast proton transfer and a large KIE on the I→A relaxation. Indeed taking the value of Kennis et al. of 400 ps for I→A for avGFP in H_2_O^3^ we determine a KIE for avGFP of 45±5. This is much larger than for the A*→I* ESPT and greatly exceeds the semi-classical limit of 6 expected from zero-point energy differences. This result is consistent with tunneling determining the GSPT rate. Such giant KIEs are not unprecedented, particularly in enzyme reactions, where they may be large and a strong function of coupling, the energies of donor and acceptor states and their environments.^12^ Essentially the same photocycle is seen in S205V, although the yield of the I state is smaller, consistent with the low quantum yield of the A*→I* compared with A*→A described above (Figure 2 D and Figure S3).

Some additional structural data on the proton wires is evident in Figures 2 C and D2. Most significantly a small but reproducible shift of the E222 C−O mode to lower wavenumber is observed between I* and I. This suggests that the H-bond to the E222 hydroxy group strengthens between I* and I.

These data are summarized in Figures 2 E and F, which show species associated spectra (SAS) for all states in the photocycle. SAS are obtained from global analysis of the data assuming a specific kinetic scheme (also shown).^13^ The scheme for T203V is consistent with the non-single exponential decay of A*, that is, parallel A_1_* and A_2_* states decay to form I*, its subsequent nanosecond decay to I and final single exponential decay to A. All the features discussed above are evident in the SAS. The quality of the fit was good, suggesting that no other intermediates are detectable in the photocycle (e.g. from isomerization or electron transfer reactions). The SAS for S205V (Figure 2 F) point to significant spectroscopic difference between ESPT active and inactive channels (A_1_* and A_2_*). The main bleach modes associated with the chromophore are similar, but the modes which arise from interaction with the protein are different; in particular, the complex transient at 1450 cm^−1^, associated with the E222 carboxylate modes,4c has a very different lineshape between A_1_* and A_2_*. Similarly the bimodal lineshape around 1642 cm^−1^, associated with excitation induced changes in protein spectra,4b has a different lineshape in the inactive A_1_* state. These changes reflect the role of protein–chromophore interactions on the TRIR spectra, noted elsewhere,4b and point to distinct protein–chromophore interactions in the two channels. However, after the formation of I* in S205V its decay to A follows a similar pathway to T203V and avGFP.

Finally, kinetics of the I→A GSPT are compared in Figure 3. This final step in the photocycle has remarkably similar time constants in all three samples (Figure 3, Table 1) with avGFP and S205V having essentially the same I state relaxation time. This is surprising, given that they have very different proton wires, causing the rate of ESPT to differ by a factor of up to 30. The most obvious explanation is that the three GSPT reactions have a common rate-determining step. Since we established that the terminal acceptor is E222 we speculate that the common rate-determining step is the initial proton tunneling from E222 injecting the proton into the wire, from which faster relaxation occurs to the A state (in this connection we note that the relevant O−O distances in the three X-ray structures are very similar). This conclusion further suggests that the proton wire in the S205V I state is stable for tens of nanoseconds (otherwise its formation would be rate-determining, and different to that of the other proteins). Recent MD simulations for avGFP and S205V A states suggested a stable proton wire for avGFP but quite frequent (ca. 0.25 ns^−1^) exchange for S205V.^14^ This simulation result is consistent with the assignment (above) of fast radiationless decay in S205V to proteins with an unformed proton wire. In contrast, the present result also suggests the proton wire is stable for several tens of nanoseconds in the S205V I state, supporting nanosecond I→A GSPT.

**Figure 3:**
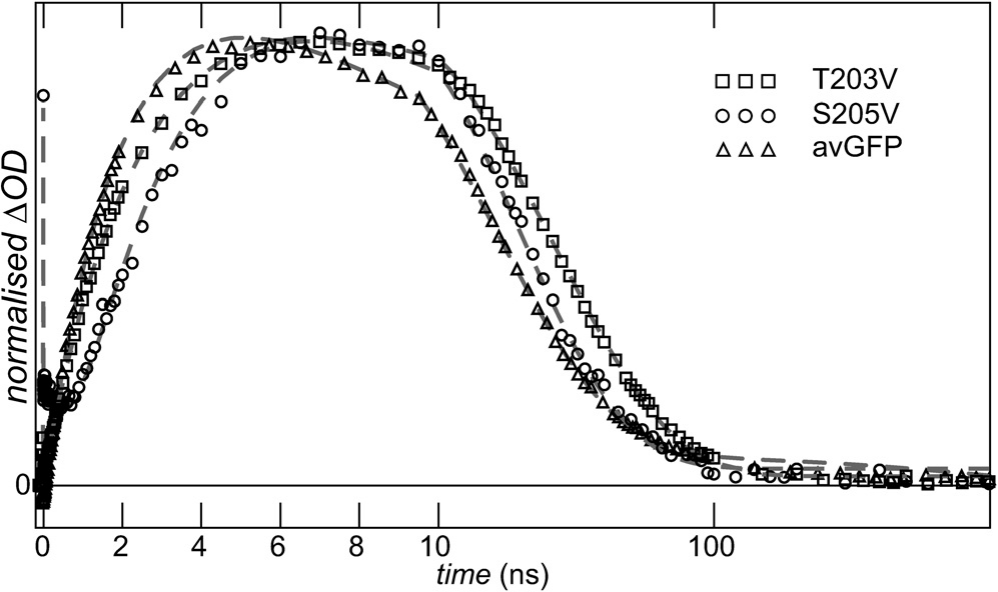
Kinetics of I→A reaction. Data are shown for all three samples studied. Dashed lines are for the global fit. Note that time axis is linear until 10 ns and a log scale later.

**Table 1:** Relaxation times associated with the states shown in Figure 2 E,F.^[a]^

	avGFP	T203V	S205V
A1^*^	13 ps	15 ps	38 ps
A2^*^	100 ps	125 ps	730 ps
I^*^	2.1 ns	2.6 ns	2.5 ns
I	17 ns	26 ns	17 ns

[a] Note that S205V has a distinct relaxation pathway (A^*^→A); the origin of this assignment is described in the Supporting Information (Figure S6).

In summary, the complete photocycles of avGFP and two key mutants have been measured with IR spectral resolution. Spectral data reveal that E222 is the acceptor in all cases, and that the distinct kinetics associated with different proton wires are associated with changes in H-bond strength and structure. A relatively low yield of ESPT in S205V reflects equilibrium between ESPT active and inactive populations of A, with the inactive form’s A* state being highly quenched. In contrast the GSPT which completes the photocycle is a tunneling reaction which has a very similar rate among the three proteins, assigned to a common rate-determining step.

## Supporting Information

As a service to our authors and readers, this journal provides supporting information supplied by the authors. Such materials are peer reviewed and may be re-organized for online delivery, but are not copy-edited or typeset. Technical support issues arising from supporting information (other than missing files) should be addressed to the authors.

miscellaneous_information
