# Supplementary material for: Complete Proton Transfer Cycle in GFP and Its T203V and S205V Mutants
Source: Angew Chem Int Ed Engl. 2015 Jun 18;54(32):9303–7. doi: 10.1002/anie.201503672 (PMC4576823; doi:10.1002/anie.201503672)
Supplement: Supplementary file 1 — miscellaneous_information [file anie0054-9303-sd1.pdf]

Angewandte Chemie
Eine Zeitschrift der Gesellschaft Deutscher Chemiker

Supporting Information

Complete Proton Transfer Cycle in GFP and Its T203V and S205V Mutants**

Sergey P. Laptenok, Andras Lukacs, Agnieszka Gil, Richard Brust, Igor V. Sazanovich, Gregory M. Greetham, Peter J. Tonge, and Stephen R. Meech**

anie_201503672_sm_miscellaneous_information.pdf

Supporting Information

Index

Experimental Details

Figure S1 Structure of S205V in the vicinity of the proton wire

Figure S2 Ultrafast time resolved IR data for avGFP

Figure S3 Picosecond kinetics of E222 Protonation

Figure S4 Fast Ground State Recovery in S205V

Figure S5 Comparison of transient IR spectra in the B state and the I state

Figure S6 Analysis procedure and assignment of kinetic scheme

References

Experimental Details

The femtosecond to millisecond time resolved IR measurements were measured using the recently described time resolved multiple probe spectroscopy (TRMPS) apparatus developed at the Harwell Research Complex. This has been described in detail elsewhere,[1] but essentially allows the measurement of high signal-to-noise (<10 μOD) IR difference spectra with <100 fs time resolution at delay times between 100 fs and 1 ms after electronic excitation of the sample. In the present experiments excitation was at 400 nm with pulses at 1 kHz of < 1 mJ energy and 100 fs width in a 100 micron radius spot size. The sample was 1 mM protein concentration in 20mM sodium phosphate and 300 mM NaCl D_2O buffer at pD 7.5 (or pH 7.5 for the aqueous measurements) contained in a 50 micron thick flow cell, which was itself rastered in the beam such that a fresh sample was illuminated by each pulse and the entire sample volume was exchanged in each raster cycle. The temperature was 295 K.

The time resolved fluorescence experiment has been described in detail elsewhere,[2] and measures fluorescence up-conversion data with sub 50 fs temporal resolution. The excitation source is a 6 mW 80 MHz beam of sub 50 fs pulses at 400 nm. The sample concentration was ca 50 μM in the pD 7.5 buffer.

Figure S1 The structure of S205V (2QLE pdb) with the proposed ESPT pathway shown

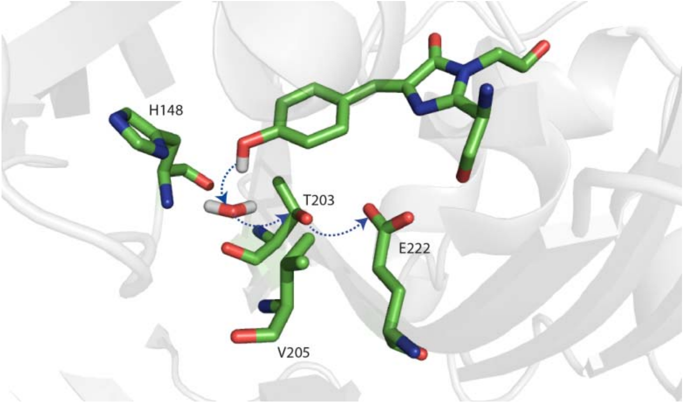

Figure S2 Ultrafast time resolved IR data for avGFP compared with T203V and S205V. A. A comparison of the DAS for avGFP (solid) and T203V (dashed), showing essentially identical kinetics and spectra, aside from the final step; B A pictorial representation of the kinetics for each of the three proteins studied; C. A comparison of the actual TRIR spectra for all three proteins, with key peaks marked.

A

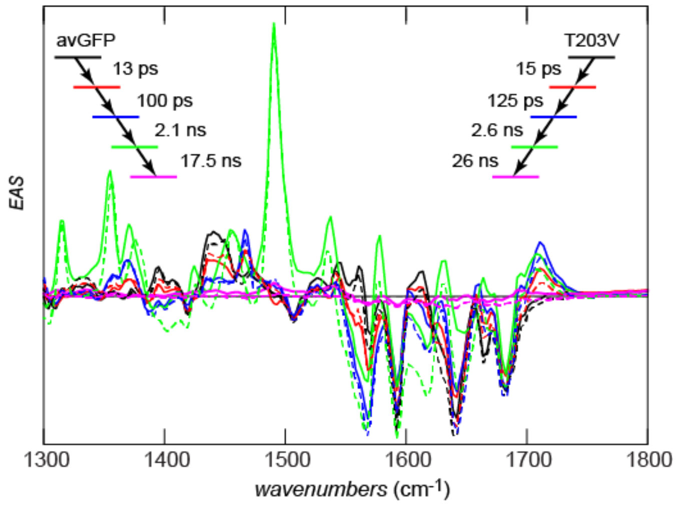

B

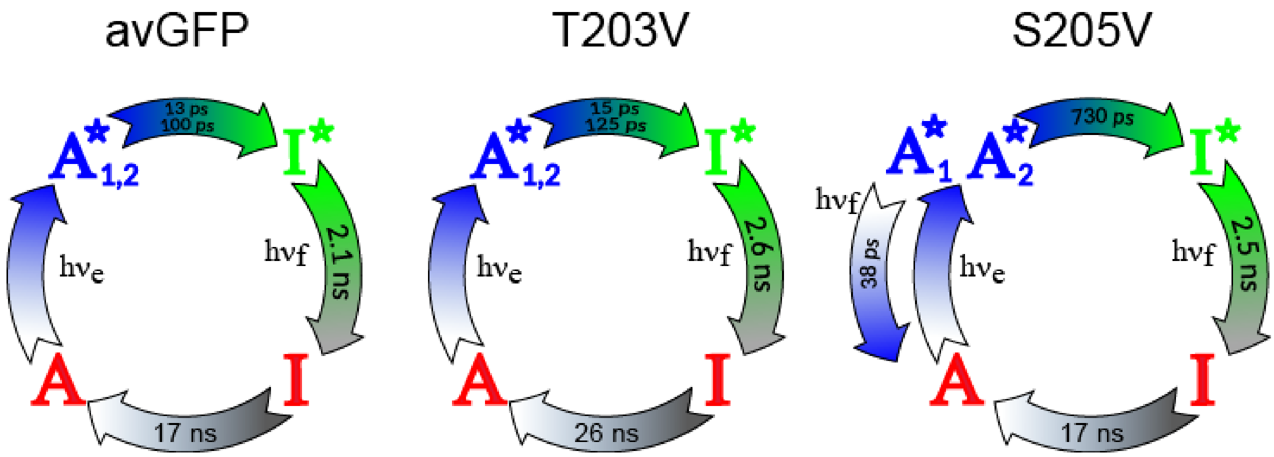

C

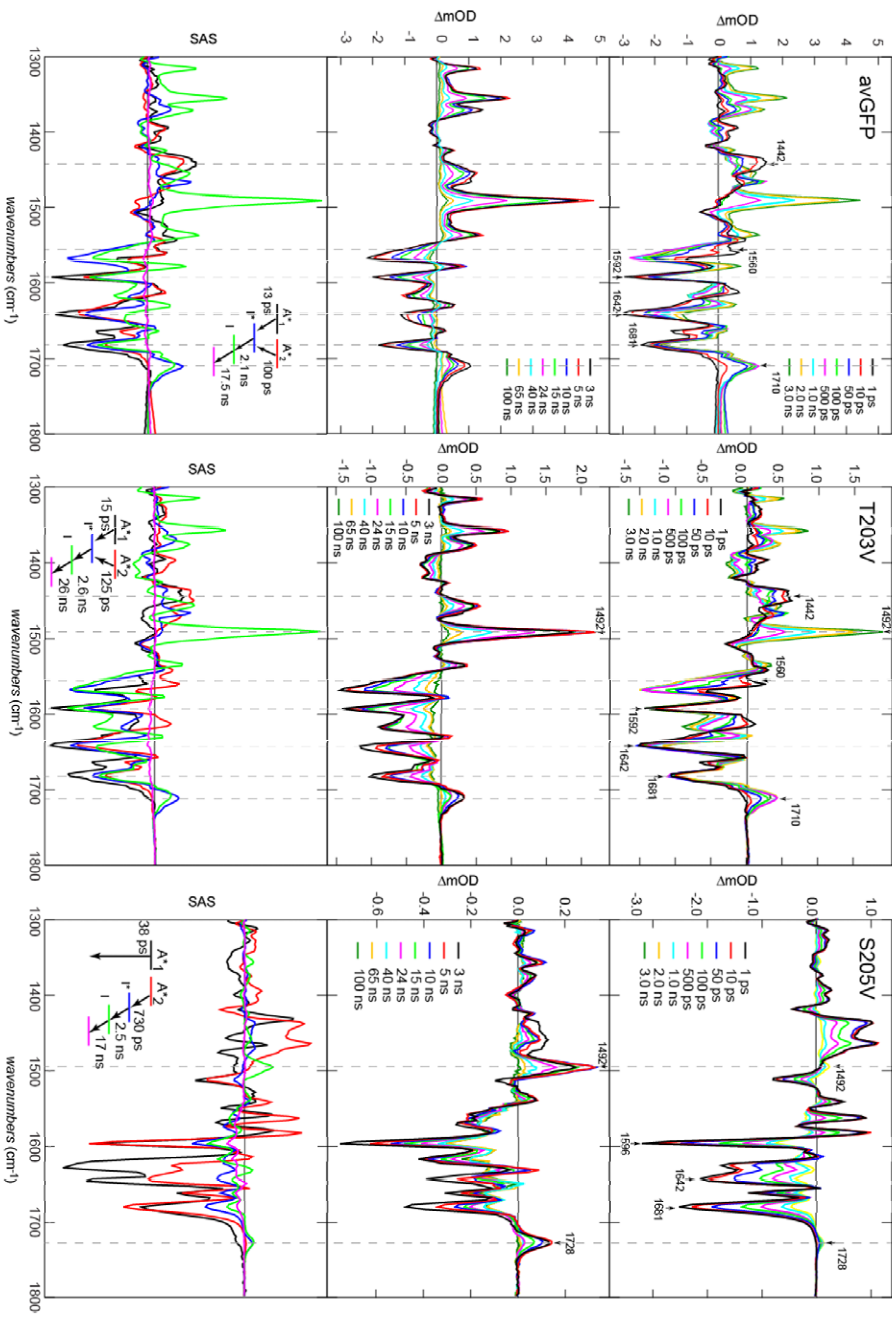

avGFP
T203V
S205V
ΔmOD
SAS
wavenumbers (cm-1)
1 ps
10 ps
50 ps
100 ps
500 ps
1.0 ns
2.0 ns
3.0 ns
3 ns
5 ns
10 ns
15 ns
24 ns
40 ns
65 ns
100 ns

Figure S3 Picosecond kinetics of E222 Protonation. The signal rises in tens of picoseconds in T203V (and avGFP), but several hundred picoseconds in S205V, before decaying on the nanosecond timescale. This is consistent with fluorescence data.

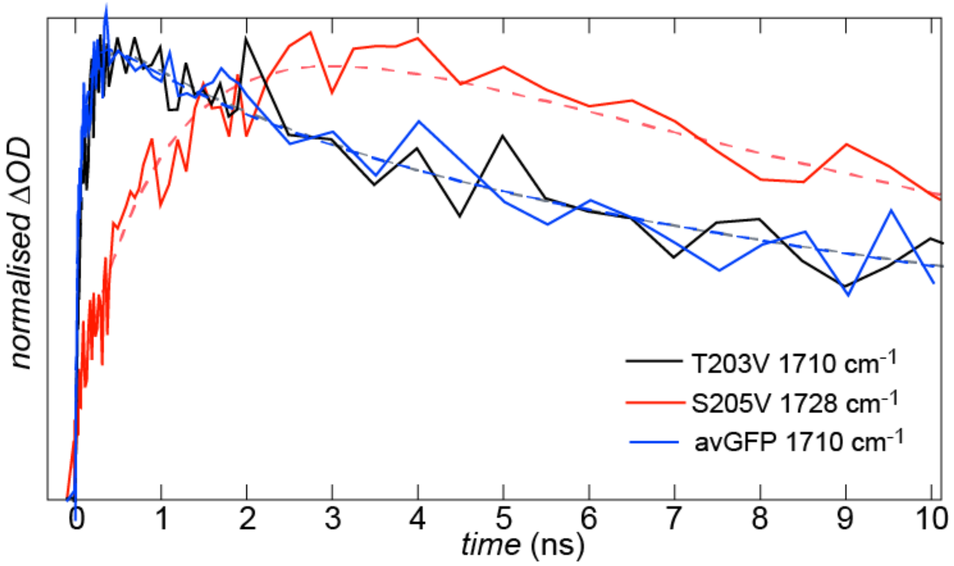

Figure S4 Fast Ground State Recovery in S205V. A The time dependence of the ground state (A state) recovery measured at the intense high frequency phenyl ring mode bleach is shown; S205 has a much faster component in its recovery. Note the linear scale is used to 10 ns and a log scale thereafter. The relevant wavenumbers are shown on the plot.

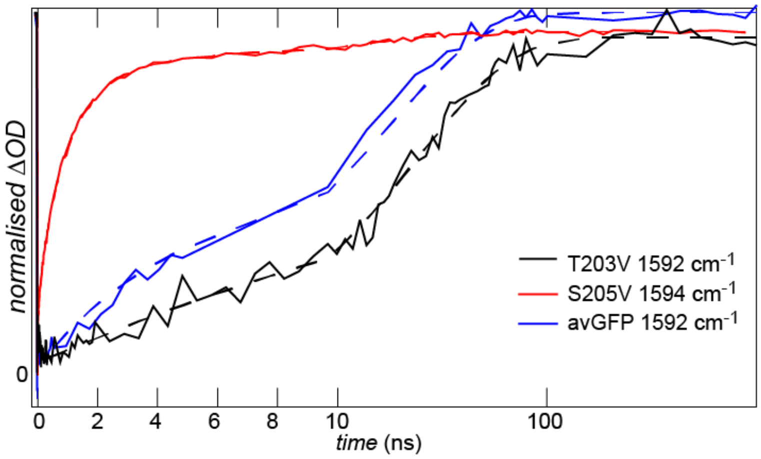

B. The transient fluorescence of S205V measured in the A* emission at 470 nm, the kinetics are independent of wavelengths and do not have a measureable isotope effect, suggesting that proton transfer is not involved in this ultrafast component. The decay times and weights are shown on the figure. The 0.2 ps decay is partly due to detected pump scatter but solvation dynamics or vibrational relaxation may also contribute. The 9 ps decay is faster than the rise observed in the I* emission, consistent with an origin in some alternative excited state decay channel rather than proton transfer. The slower component (close to our upper time resolution) is consistent with the time scale for A*→ I* ESPT observed in pico-nanosecond time resolved fluorescence and transient IR.

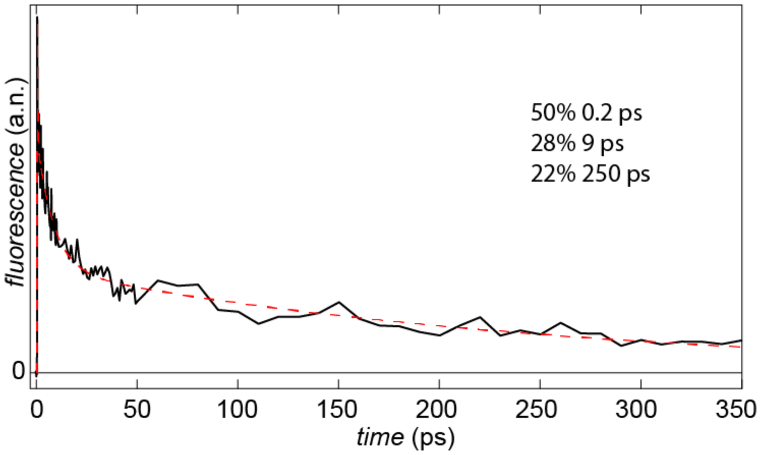

Figure S5 Comparison of transient IR spectra in the B state and the I state. The transient IR of the B state of a GFP mutant trapped in that state (S65T at pD 8) is compared with the microsecond delayed time resolved IR of T203V and S205V. The correspondence of the S65T B state bleach and the (inverted for comparison) T203V transient absorption is apparent for at least 3 modes, confirming that the I state kinetics are indeed monitored at this wavenumber. Differences between the two spectra reflect both a greater contribution from perturbed protein modes in the T203V/S205V spectra (as expected because the protein will be perturbed by the new charge distribution) and the different mutations.

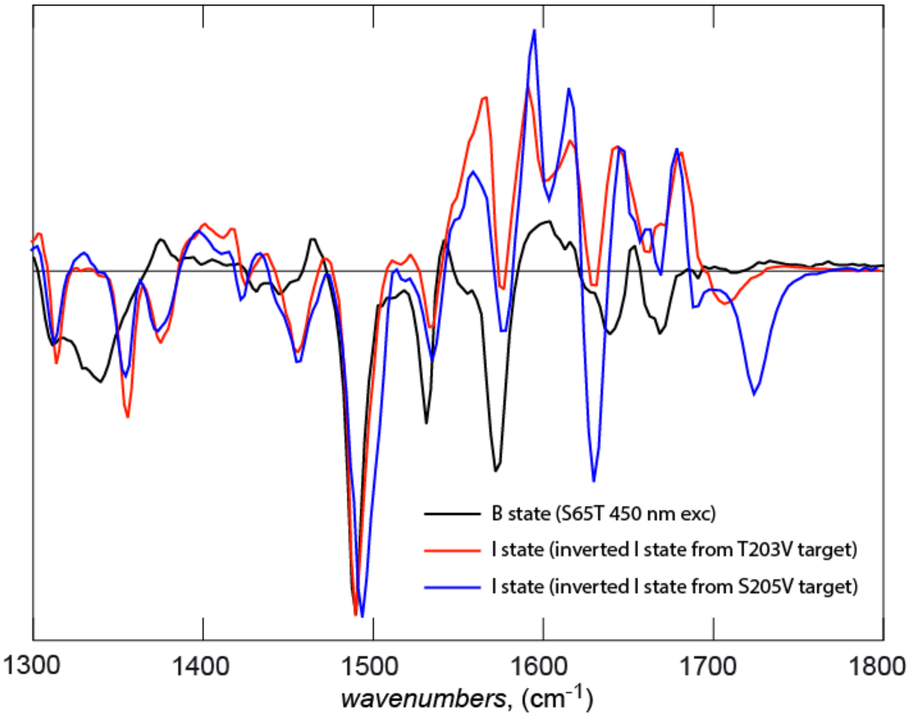

Figure S6 Analysis procedure and assignment of kinetic schemes. The global analysis procedure simultaneously fits all kinetic traces at all wavenumber to a number of parallel exponentially decaying components, which results in decay associated spectra (DAS); these DAS represent estimated spectral amplitudes of each kinetic component. In most cases DAS are a pure mathematical decomposition of the data that does not represent real physical/chemical species. However the sign of the DAS can be an indicator of decaying or rising signals. For both avGFP and T203V we can see a negative region around 1710 cm^{-1} in the first two DAS (black and red) with exactly the same shapes. This can be interpreted as a bi-exponential rise of the signal due to protonated E222 in that region. In contrast in S205V we see only one (red) DAS negative around 1730 cm^{-1}, while the first (black) DAS has no amplitude there. This difference can be interpreted as a pseudo mono-exponential rise of the signal in that region on the timescale of 730 ps (consistent with the mean rise time of I* seen in time resolved fluorescence) while on the 38 ps time of the black DAS there is no evidence for protonation of E222; this component is associated in our kinetic scheme with the A*→A ground state recovery seen in time resolved fluorescence (Fig S4).
Negative features of the blue DAS in the same regions in all experiments on the timescale of ca 2 ns can be attributed to a shift of the corresponding peak, indicative in the E222 case of a change in H-bond strength, as described in the main text.

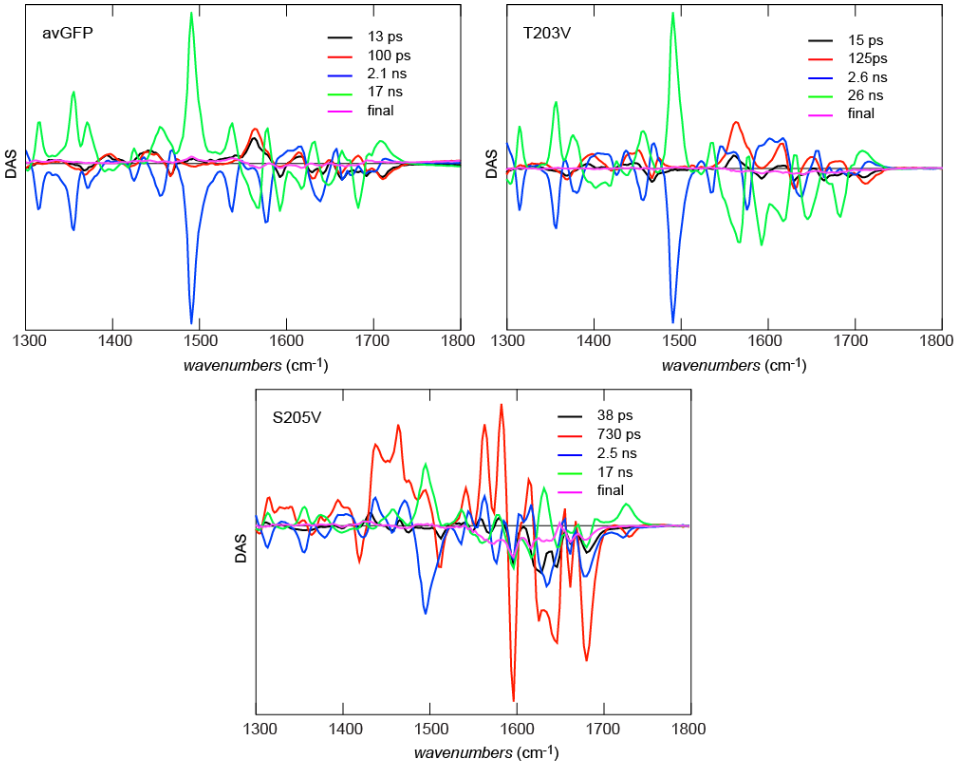

References

[1] G. M. Greetham, D. Sole, I. P. Clark, A. W. Parker, M. R. Pollard, M. Towrie, *Rev. Sci. Instrum.* **2012**, *83*.

[2] I. A. Heisler, M. Kondo, S. R. Meech, *J. Phys. Chem. B* **2009**, *113*, 1623-1631.
